# Reduced phosphorus intake throughout gestation and lactation of sows is mitigated by transcriptional adaptations in kidney and intestine

**DOI:** 10.1186/s12864-020-07049-0

**Published:** 2020-09-11

**Authors:** Aisanjiang Wubuli, Christian Gerlinger, Henry Reyer, Michael Oster, Eduard Muráni, Nares Trakooljul, Siriluck Ponsuksili, Petra Wolf, Klaus Wimmers

**Affiliations:** 1 Leibniz Institute for Farm Animal Biology (FBN), Wilhelm-Stahl-Allee 2, 18196 Dummerstorf, Germany; 2 Nutritional Physiology and Animal Nutrition, University of Rostock, Justus-von-Liebig-Weg 6b, 18059 Rostock, Germany; 3 Animal Breeding and Genetics, University of Rostock, Justus-von-Liebig-Weg 7, 18059 Rostock, Germany

**Keywords:** Dietary phosphorus, Mineral requirement, Monogastric farm animals, Phosphorus homeostasis, Gestational diets, Lactating sows

## Abstract

**Background:**

The environmental impact of pig farming need to be reduced, with phosphorus (P) being of particular interest. Specified dietary regimens and management systems contribute to meet environmental concerns and reduce economic constrains. However, pregnant and lactating sows represent vulnerable individuals, whose reproductive potential and metabolic health status relies on adequate supply of macro- and micronutrients. The aim of this study was to investigate, whether sows fed with a dietary P content that is below or above current recommendations are capable to maintain mineral homeostasis during the reproduction cycle and which endogenous mechanisms are retrieved therefore in kidney and jejunum. Nulliparous gilts were fed iso-energetic diets with recommended (M), reduced (L), or high (H) amounts of mineral P supplements throughout gestation and lactation periods. Blood metabolites and hormones referring to the P homeostasis were retrieved prior to term (110 days of gestation) and at weaning (28 days of lactation). Transcriptional responses in kidney cortex and jejunal mucosa were analyzed using RNA sequencing.

**Results:**

The variable dietary P content neither led to an aberration on fertility traits such as total weaned piglets nor to an effect on the weight pattern throughout gestation and lactation. Serum parameters revealed a maintained P homeostasis as reflected by unaltered inorganic P and calcium levels in L and H fed groups. The serum calcitriol levels were increased in lactating L sows. The endocrine responses to the dietary challenge were reflected at the transcriptional level. L diets led to an increase in *CYP27B1* expression in the kidney compared to the H group and to an altered gene expression associated with lipid metabolism in the kidney and immune response in the jejunum.

**Conclusions:**

Our results suggest that current P requirements for gestating and lactating sows are sufficient and over supplementation of mineral P is not required. Shifts in renal and jejunal expression patterns between L and H groups indicate an affected intermediate metabolism, which long-term relevance needs to be further clarified.

## Background

Phosphorus (P) is present in every cell in the body and is involved in numerous biological processes like bone mineralization, energy metabolism, and intracellular signaling [1]. Consequently, P is also important for growth processes and bone development in livestock, whereby practically often more P is supplied in dietary formulations than is required for age-specific processes [2, 3]. An inefficient utilization and the over supplementation of inorganic P may not only interfere with the mineral homeostasis of the organism [4], but can also lead to imbalances in the agricultural cycle through excessive nutrient excretion [5, 6]. Therefore, an efficient utilization of dietary supplied P sources is essential for the sustainable use of the non-renewable resource P and the maintenance of an adequate organismal P homeostasis. Since monogastric livestock species, including pigs, account for the majority of global meat production, strategies for the efficient use of inorganic and plant-based P could contribute to improving global P efficiency and achieving a sustainable P cycle.

Body P homeostasis is mainly modulated through the P absorption by the small intestine (especially the jejunum), retention/excretion of minerals in the kidney, and P storage in the bone [7]. Hormonal factors including calcitriol (1,25(OH)_2_VitD_3_) are important regulators of the organism’s P homeostasis [8]. Their regulatory activity is also being altered by the amount of dietary P intake [9]. In our previous studies, variable dietary P intake was shown to affect the expression levels of active P transporters [10], and numerous other genes involved in the regulation of mineral homeostasis with immunomodulatory effects in kidney and small intestine of growing pigs [11]. However, it is still under debate whether these effects of dietary P levels are directly sensed and mediated or indirectly orchestrated through downstream signaling cascades of the Ca sensing receptor (CaSR) [12, 13].

Previous studies have shown that P utilization also differs within and between different genetic pig breeds and thus, P homeostasis is influenced by genetics and transcriptional regulations [14–17]. Therefore, strategies towards improved P utilization should be re-evaluated considering animal-intrinsic factors such as genetics and specific metabolic requirements of modern pig breeds. To date, numerous studies have been conducted with growing pigs to investigate P utilization and its interrelation with bone metabolism and immune parameters in a period of high nutrient demand for anabolic processes [17–19]. In sows, the requirements of the organism differ considerably from those of growing pigs and are considered in the corresponding nutritional recommendations [20]. For gestating and lactating sows, the diets are formulated in order to ensure a sufficient supply of nutrients for maintenance, fetal development, and milk production [21]. Due to the metabolic changes occurring at the onset of milk production at farrowing, lactating sows can compensate by mobilizing their nutrient reserves, in particular protein or fat [22]. Thereby, small amounts of P (~ 0.97 g/d) can be mobilized to meet P requirements [23]. As known in mammals, the organism can further induce compensatory mechanisms for fetal and early postnatal P supply by increasing intestinal mineral absorption [24]. However, in general the microbial digestibility of dietary P sources in reproductive sows is lower compared to growing pigs [25], resulting in increased excretion of P into the environment [26].

A deeper understanding of the specific molecular mechanisms of P homeostasis in sows following dietary interventions could contribute to a more sustainable P utilization in pig husbandry. Gestating and lactating sows are of particular interest, as they have specific nutritional and metabolic demands and optimally utilize the available resources for the pre- and postnatal supply of the fetuses and offspring. In the current study, holistic expression patterns and blood phenotypes were analyzed to investigate the comprehensive effect of divergent levels of dietary P throughout gestation and lactation on sow physiology.

## Results

### Effects of a divergent dietary P supply on phenotype

The average body weight development and muscle characteristics in each experimental group are shown in Tables 1 and 2. The average body weight of sows at slaughtering was 201.1 ± 3.6 kg. No significant differences were observed in zoo-technical parameters and post mortem meat characteristics between the dietary groups. The different feeding had no effect on the health status of the animals.

**Table 1:** Average body weight of sows fed divergent amounts of dietary P (mean ± SE)

Body weight [kg]	L	M	H
0 day of gestation (insemination)	164.5 ± 2.9	168.7 ± 3.3	164.1 ± 4.7
30 days of gestation	193.8 ± 7.6	208.8 ± 8.0	195.0 ± 7.6
56 days of gestation	187.8 ± 8.7	177.8 ± 9.0	177.6 ± 10.7
84 days of gestation	219.4 ± 3.6	230.5 ± 5.1	220.0 ± 5.9
105 days of gestation	240.6 ± 2.8	250.5 ± 5.2	240.4 ± 7.5
28 days of lactation (weaning)	202.2 ± 5.3	212.75 ± 8.5	199.4 ± 5.4

**Table 2:** Slaughter weight and meat characteristics of sows fed divergent amounts of dietary P (mean ± SE)

Trait	L	M	H
Live weight at slaughter (kg)	201.0 ± 4.9	208.3 ± 8.8	195.4 ± 5.3
Carcass weight (kg)	127.5 ± 7.8	131.2 ± 6.1	124.1 ± 5.3
MLD pH at 45 min	6.30 ± 0.10	6.18 ± 0.10	6.34 ± 0.04
MLD pH at 24 h	5.56 ± 0.05	5.53 ± 0.07	5.51 ± 0.03
MSM pH at 45 min	6.17 ± 0.19	5.89 ± 0.17	6.02 ± 0.15
MSM pH at 24 h	5.53 ± 0.05	5.51 ± 0.05	5.53 ± 0.06
MLD ash (%)	1.09 ± 0.01	1.11 ± 0.01	1.12 ± 0.01

*MLD Musculus longissimus dorsi*, *MSM Musculus semimembranosus*

**Fig. 1:**
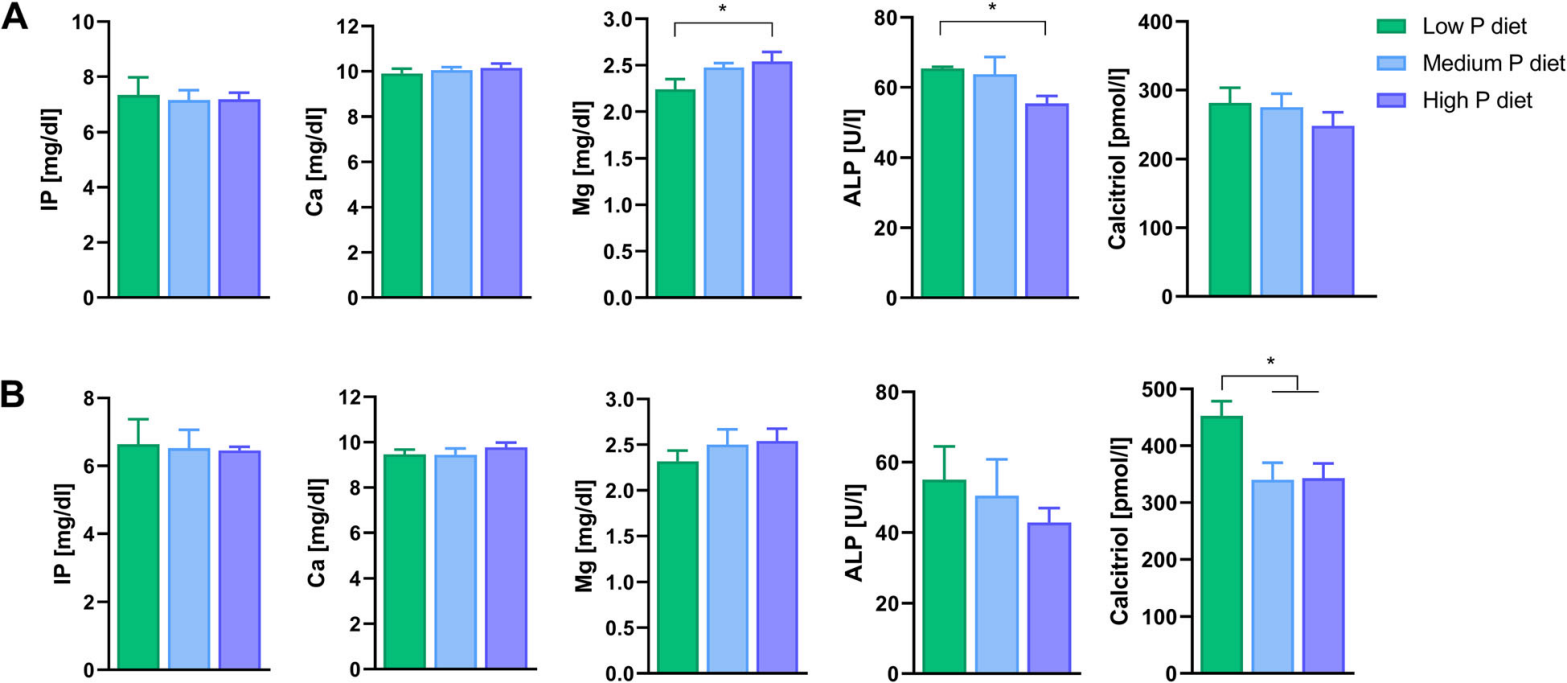
Serum analytics of sows fed diets containing low, medium and high dietary P contents throughout gestation and lactation. Effects of divergent dietary P supply on serum inorganic P (IP), calcium (Ca), magnesium (Mg), alkaline phosphatase activity (ALP) and calcitriol (mean ± SE) at 110 days of gestation (**a**) and at 28 days of lactation (**b**). Superscripts indicate statistical significance between groups (*, *p* < 0.05)

Serum measurements comparing dietary groups are displayed in Fig. 1. Dietary treatments had no significant effects on serum inorganic P and Ca levels. However, lower alkaline phosphatase activity (*P* = 0.042) was observed in the H group compared to the group L at 110 days of gestation. A significantly higher level of serum calcitriol was observed in L fed animals compared to sows fed on M and H diets at 28 days of lactation (L vs. M, *P* = 0.029; L vs H, *P* = 0.026).

The reproductive performance of gestating sows was not impaired by modification of the dietary P supply (*P* > 0.05). The total litter size (mean ± SD) of sows was in average 15.0 ± 2.5 piglets for L, 14.8 ± 1.9 piglets for M and 13.8 ± 2.8 piglets for H. The respective average total number of born alive piglets per sow for L, M and H groups was 14.8 ± 2.5, 14.0 ± 2.2 and 13.0 ± 2.5, respectively. The average birth weights of piglets were 1.30 ± 0.27 kg (L), 1.25 ± 0.33 kg (M) and 1.22 ± 0.30 kg (H).

### Effects of a divergent dietary P supply on transcriptional profiles in kidney and jejunum

**Fig. 2:**
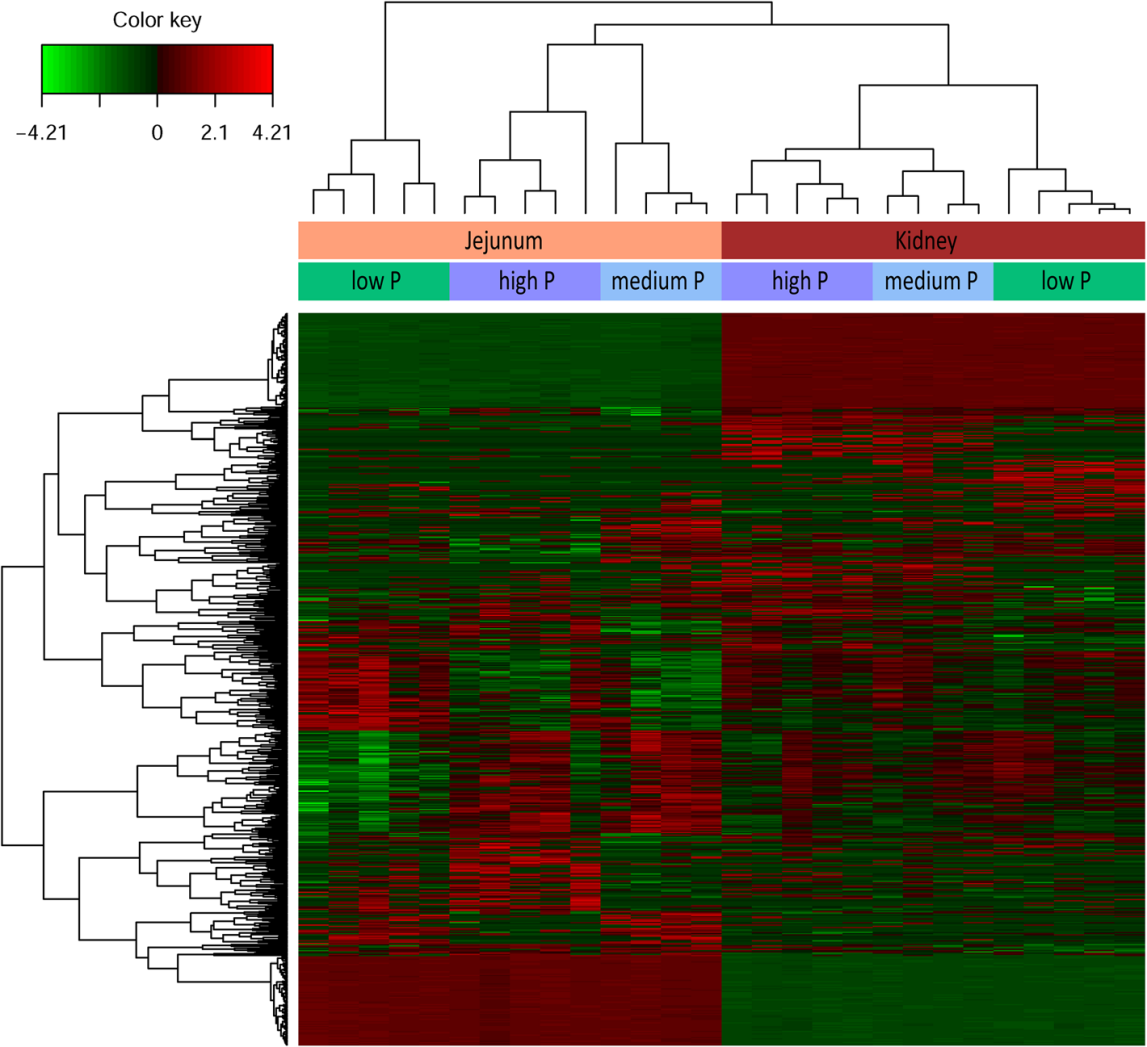
Hierarchical clustering based on expression profiles from jejunum and kidney tissue of sows fed diets containing low, medium and high dietary P contents throughout gestation and lactation. The colors inside the heatmap indicate up regulation (red) and down regulation (green) of genes

A total of 28 RNA libraries from the kidney and jejunum of 14 sows fed medium (*n* = 4), lower (*n* = 5) and higher (*n* = 5) level of dietary P were sequenced and analyzed. Mapping of the processed sequences to the reference genome yielded per sample approximately 56 and 68 million high quality paired-end reads in kidney and jejunum, respectively. The average mapping efficiency was 98.5%. After filtering of absent and very low abundant genes, a total of 17,142 genes (13,995 annotated) in kidney and 16,693 genes (13,644 annotated) in jejunum were identified. To get an overview of the expression profile, a hierarchical clustering analysis of the selected variables (sPLS-DA) was performed (Fig. 2). Overall, the tissue effect dominated, which results in a clear separation of individual jejunum and kidney samples. Within each tissue, the expression profiles of the individuals of M and H groups showed lower distance than those of the L group.

**Fig. 3:**
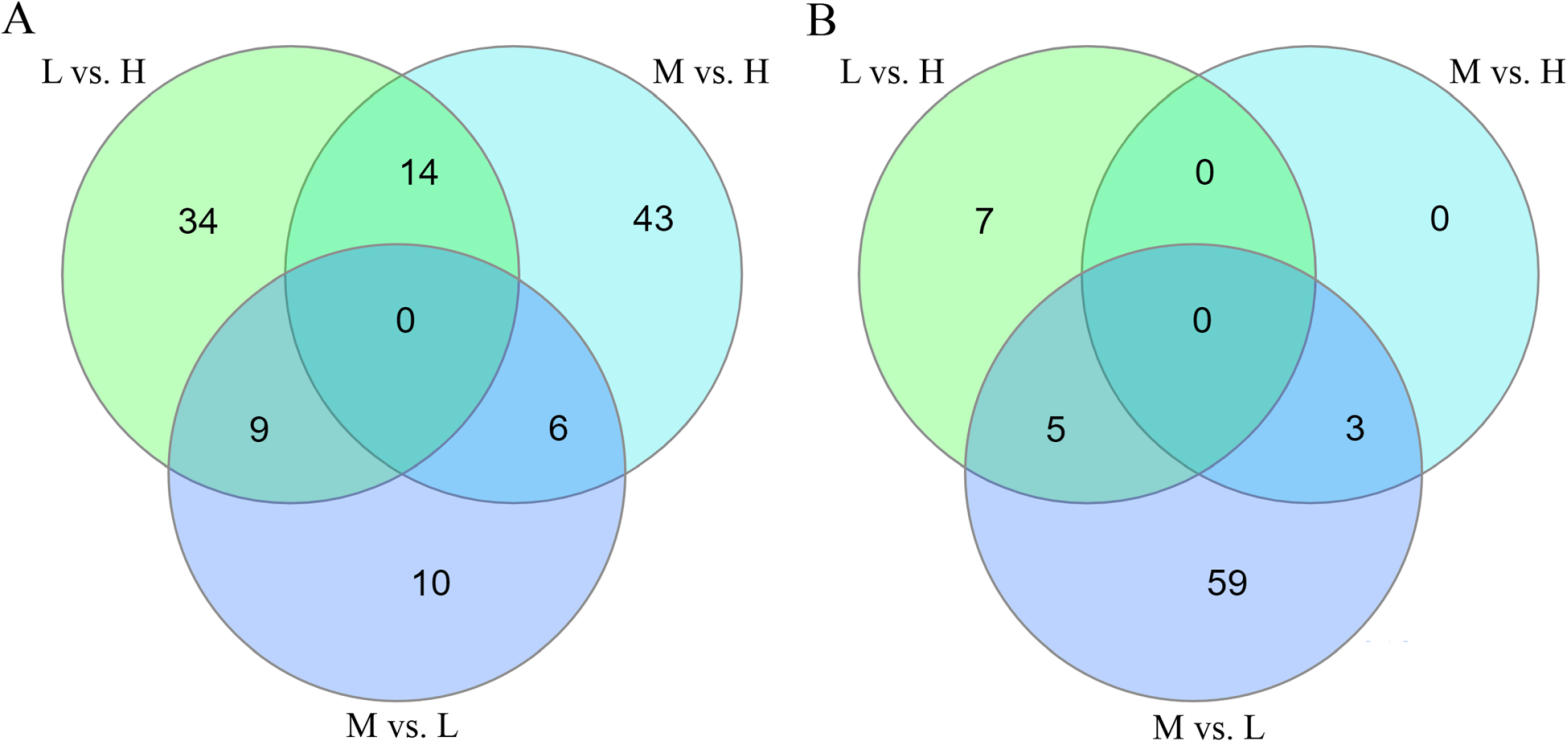
Venn diagram of DEGs in the kidney (**a**) and the jejunum (**b**) of sows fed diets containing low (L), medium (M) and high (H) dietary P contents throughout gestation and lactation. L vs. H: comparison between group L and H; M vs. H: comparison between group M and H; M vs. L: comparison between group M and L

In kidney, 57 genes (55 annotated; L vs. H), 63 genes (53 annotated; M vs. H) and 25 genes (24 annotated; L vs. M) were differentially expressed between sows (q ≤ 0.10 corresponding to *P* ≤ 0.001). In jejunum, 12 genes (L vs. H), three genes (M vs. H) and 67 genes (64 annotated, L vs. M) were altered significantly between groups (q ≤ 0.10 corresponding to *P* ≤ 0.001). The overlap of differently expressed genes between the different comparisons in the two tissues is shown in Fig. 3. The full list of differentially expressed genes is shown in Supplementary Table S1.

The complete list of DEGs in kidney and jejunum was subjected to KEGG pathway analysis to identify significantly affected biological pathways. Sixteen canonical pathways were observed among DEGs in kidney, of which the highest significantly enriched pathways are “metabolic pathways” (18 DEGs), “PPAR signaling pathways” (7 DEGs), “Protein processing in endoplasmic reticulum” (5 DEGs), “Fatty acid metabolism” (5 DEGs) and “Fatty acid degradation” (4 DEGs). In the jejunum, top three pathways, “metabolic pathways” (13 DEGs), “Viral protein interaction with cytokine and cytokine receptor” (3 DEGs) and “IL-17 signaling pathway” (3 DEGs) were revealed.

## Discussion

In order to gain a deeper understanding of the molecular mechanisms of P utilization in sows, respective blood parameters and transcriptional responses to divergent dietary P levels were investigated in this study. Concerning phenotypic characteristics, significant differences in serum ALP activity and calcitriol levels between the L and H groups were observed in gestating and lactating sows. The mRNA patterns illustrate the tissue-specific effects of dietary P and its contribution to maintain P homeostasis.

### Effects of dietary P supply on the expression of renal genes involved in P homeostasis and lipid metabolism

The KEGG pathway analysis of DEGs in the kidney revealed 16 significantly affected pathways. A total of 18 genes including the *CYP27B1 (cytochrome P450 family 27 subfamily B member 1)* were enriched in the “metabolic pathways” (Table 3). *CYP27B1* is significantly up-regulated in L compared to H (FC: 2.4, L > H). Renal *CYP27B1* is regulated by hormones like parathyroid hormone (PTH) and fibroblast growth factor 23 (FGF23), and is induced by a reduced availability of P [9, 27]. In fact, *CYP27B1* encodes an enzyme called 1-alpha-hydroxylase, which is responsible for the conversion of the storage form of vitamin D_3_ (calcidiol, 25(OH)VitD_3_) to the active form calcitriol (1,25(OH)_2_VitD_3_). Consequently, serum measurements showed significantly higher levels of calcitriol in the blood of L animals compared to M and H at 28 days of lactation. Calcitriol is important for maintaining the proper balance of Ca and P in the body by affecting intestinal absorption, renal excretion and bone remodeling [28, 29]. Despite the increased mineral requirements during pregnancy and lactation, serum analyses in this study suggest that L sows are capable to maintain P homeostasis via endogenous mechanisms and to control serum levels of Ca and P and the respective Ca:P ratio within a narrow range [30].

**Table 3:** Significantly enriched canonical pathways in kidney and jejunum of sows fed diets containing low, medium, and high dietary P contents throughout gestation and lactation

Tissue	KEGG pathway	Number of annotated genes in the pathways	FDR-adjusted ***P***-value	Involved genes
**Kidney**	Metabolic pathways	1388	1.38E-04	GALT,ENTPD5,ACADVL,MAT2A, ACSL1,ACOX2,CBR1,MGLL, DGAT2,G6PC,GSTA1,ACSM4, ACAA1,ALDH3A1,CYP27B1, HAO1,FADS2,BDH1
	PPAR signaling pathway	75	2.68E-06	CPT2,ACOX2,ACSL1,ACAA1, FABP3,FADS2,SLC27A2
	Protein processing in endoplasmic reticulum	161	6.72E-03	HSPA8,DNAJB1,DNAJA1,HSPH1, HSPA5
	Peroxisome	84	5.78E-04	ACOX2,HAO1,ACAA1,ACSL1, SLC27A2
	Fatty acid metabolism	57	1.38E-04	ACADVL,ACSL1,ACAA1,FADS2, CPT2
	Protein digestion and absorption	86	6.72E-03	COL3A1,SLC7A7,COL1A2,COL1A1
	Fatty acid degradation	41	5.78E-04	ACSL1,ACAA1,ACADVL,CPT2
	Metabolism of xenobiotics by cytochrome P450	49	1.20E-02	ALDH3A1,CBR1,GSTA1
	Complement and coagulation cascades	80	3.10E-02	C1S,PLG,C7
	ECM-receptor interaction	84	3.16E-02	COL1A2,FN1,COL1A1
**Jejunum**	Metabolic pathways	1388	3.58E-03	GALK1,B3GALT2,HMOX1,DHRS4,NT5E,XDH,IDUA,FUT2,PHOSPHO1,ADA,CHPF,SPR,PLA2G2D
	Viral protein interaction with cytokine and cytokine receptor	84	4.38E-02	CXCL9,CCL20,CXCL10
	IL-17 signaling pathway	88	4.38E-02	CCL20,MAPK6,CXCL10

Interestingly, the *STC1 (Stanniocalcin 1)* gene, a glycoprotein hormone, was down-regulated in group H compared to M (FC: 1.83). It has been demonstrated that the expression of *STC1* is induced by circulating Ca [31], calcitriol and PTH [32] in the kidney, and that *STC1* stimulates P absorption in the small intestine and reabsorption in the proximal tubules of the kidney [33]. This suggests that a high intake of dietary P induces molecular mechanisms that counteract an oversupply with Ca and P of the organism. Due to the absence of regulations at the level of transcellular P transporters, this might mainly involve actions on paracellular transport processes in kidney and intestine. At the physiological level, a recent study on growing piglets also showed that an increased dietary P intake has no further beneficial effects on bone tissue synthesis [34]. Overall, the feeding recommendations for sows seem to exceed the current requirements.

In kidney, a group of genes associated to “metabolic pathways”, including *ACOX2 (branched chain acyl-CoA oxidase), ACSL1 (Long-chain Acyl-CoA synthetase-1)*, *ACADVL (Long chain acyl-CoA dehydrogenase), ACAA1 (acetyl CoA acyltransferase 1)* and *CPT2 (carnitine palmitoyltransferase 2)* is also associated with “PPAR signaling pathway”, “peroxisome” and “fatty acid metabolism/degradation”. Indeed, all genes mentioned above are involved in the β-oxidation of fatty acids, which is the major pathway for fatty acid degradation.

Previous studies in rodents suggested that a high P diet can affect tissue-specific energy metabolism by hampering lipid synthesis, while increasing the expression of genes associated with lipid oxidation [35, 36]. However, in our study on pregnant and lactating sows, all genes related to β-oxidation mentioned above were more abundant in the kidney of L compared to H animals. Consequently, it can be deduced that the renal up-regulation of these genes under reduced dietary P supply could reflect the fact that energy is locally provided by increased β-oxidation for the function of the kidney cells i.e. to retain P in the organism. However, the NCBI gene databases [37] showed that all these genes related to fatty acid metabolism are highly expressed in many organs and tissues including kidney. Considering the fact that lactating sows can mobilize their nutrient reserves (primarily fat) for milk production, the observed differences between L and H groups might indicate a metabolic shift towards energy supply from lipids via β-oxidation in L animals. In fact, other studies clearly showed that fatty acid metabolism changes significantly as gestation progresses [38], and sows gain excessive fat during gestation to be mobilized in lactation [39]. This metabolic change could also increase the rate of mortality at birth [40], but there is no evidence for this in the current study.

In addition, a group of DEGs including the heat shock proteins (HSPs) *HSPA5, HSPA8, HSPH1, DNAJA1* and *DNAJB1* was higher abundant in L group compared to H animals, and is enriched in the KEGG pathway “protein processing in endoplasmic reticulum (ER)”. The HSPs act as molecular chaperones that exert a critical role in protein homeostasis by preventing the aggregation of un−/misfolded proteins and cell death in stress conditions [41, 42]. The ER is also the place of phospholipid synthesis and storage of Ca, and many other protein-folding chaperones in the ER require a high level of Ca for their work [43]. Obviously, the maintenance of mineral homeostasis for L sows at the cellular level might be considered as a stressor, which effects should be assessed across tissues in further studies. In addition, several genes associated with the extracellular matrix formation including the collagen type *COL1A1 (type I collagen alpha 1), COL1A2 (type I collagen alpha 2)* and *COL3A1 (type III collagen alpha 1)*, which are known to be involved in the “Protein digestion and absorption” pathway were higher abundant in H compared to L. The *COL1A1* and *COL1A2* genes jointly produce a large molecule called type I collagen, which is known as a key protein involved in bone density, mineralization and development [44]. Interestingly, it has been demonstrated that high P diet induced collagen fibril organization and caused fibrosis in rat kidney [45]. Furthermore, *FMOD (fibromodulin)* and *FN1 (fibronectin 1)* were significantly down-regulated in L compared to the other groups. Fibromodulin plays a role in bone mineralization, and its deficiency causes osteoporosis [46]. Fibronectin, an extracellular matrix protein, plays an essential role in the initiation and progression of fibrillogenesis through interaction with other fibronectin molecules and extracellular matrix components such as collagens [47]. Thus, this might indicate that a reduced dietary P level could prevent the excessive accumulation of extracellular matrix proteins (e.g. collagen, fibronectin) and fibrogenesis in the kidney.

Moreover, one member of the transmembrane water and small solute channels, *AQP11* (*Aquaporin 11*), was significantly down-regulated in the high P group (H < M, H < L). The human orthologue (h*AQP11)* is found to be localized in the adipocytes and function as both water and glycerol channel [48]. However, the exact role of *AQP11* in the kidney, whether it transports only water or also other molecules like glycerol, is unclear [49]. Further, several members from the solute carrier (SLC) family were significantly altered between the two extreme dietary groups. The *SLC44A4 (Thiamine Pyrophosphate Transporter)*, *SLC5A9 (Sodium/Glucose Cotransporter)*, *SLC26A6 (Anion Transporter)* and *SLC4A1 (Anion Exchanger)* were higher abundant in the H group, while the *SLC16A13 (Monocarboxylic Acid Transporters)* and *SLC25A45 (acyl carnitine transporter)* were higher abundant in L.

### Effects of dietary P supply on gene expression in jejunum

Differentially expressed genes in jejunum revealed three canonical pathways (Table 3). A group of genes including the *PHOSPHO1* and *NT5E* genes was shown to be associated with the “metabolic pathways”. *PHOSPHO1* is a phosphatase and was down-regulated in group L compared to M. It has been reported that the gene product of *PHOSPHO1* is involved in providing inorganic P for cartilage and matrix mineralization and in the initiation of calcification processes [50, 51]. Thus, a reduced P diet might prompt adaptations at peripheral tissue sites to preserve the extracellular mineral pool. The *NT5E* (5′-Nucleotidase Ecto) gene is down-regulated in the M group compared to L and H (M vs. H, FC: − 4.92; M vs. L, FC: − 3.97). This gene encodes CD73, which hydrolyzes extracellular adenosine monophosphate into adenosine and inorganic phosphate, and plays a role in the inhibition of ectopic tissue calcification [52] and cellular immune response [53]. The *CXCL9, CCL20 and CXCL10* genes were shown to be associated with the canonical pathway of “Viral protein interaction with cytokine and cytokine receptor”. Moreover, *CCL20, MAPK6* and *CXCL10* were shown to be associated with the canonical pathway of “IL-7 signaling pathways”. Considering the differential abundance of these genes, the observations might indicate that a high P diet has an inhibitory effect on the immune response in the jejunum. In previous studies, similar immune changes were also observed in the jejunum [11, 54, 55].

The *SLC10A2 (Sodium/Bile Acid Cotransporter)* and *CPT1A (carnitine palmitoyl transferase 1a)* genes had a significantly higher mRNA abundance in group H compared to L. It has been reported that the *SLC10A2* encoded transporter is essential for intestinal reabsorption of bile acid, which is associated with cholesterol/lipid metabolism [56, 57]. The *CPT1A* gene is also suggested as a key player in lipid metabolism due to its role in β-oxidation of long-chain fatty acids [58]. Comparing the M and the H group, the *PON3 (paraoxonase 3)* gene was significantly up-regulated in H. *PON3* was demonstrated to be associated with high-density lipoproteins [59], which play a role in the modulation of cholesterol levels in the body [60].

Deviating dietary P seems to have no significant effects on the sodium dependent P absorption in the jejunum of sows. Actually, it has been demonstrated that intestinal P absorption is mediated by trans- and paracellular transport systems and P transport is affected by factors like intestinal pH and phosphonoformic acid levels [61]. Therefore, in this study, effects of divergent levels of dietary P on the gene expression of major intestinal P transporters could be masked by other cofactors in the jejunum.

## Conclusions

We investigated the transcriptional responses in kidney and jejunum to different dietary P supplies and its correlation with the phenotypic characteristics of sows. The differential P supply of the sows throughout pregnancy and lactation showed no influence on the performance characteristics, but triggered endocrine adaptation. The transcriptional responses of kidney and jejunum showed the regulation of pathways related to protein processing in the endoplasmic reticulum, fatty acid metabolism and innate immune characteristics. Interestingly, DEGs are evident within signaling pathways that have been functionally characterized for their role in pathological processes of mineral homeostasis such as ectopic calcification, osteoporosis or fibrillogenesis. This study shows that the regulation of these genes is involved in the physiological response to slight nutritional imbalances. Taken together, intestinal and renal responses to a continuous dietary P reduction can trigger rather complicated molecular mechanisms, whereby a distinction must be made between the local provision of energy and structures and the organismic maintenance of mineral homeostasis.

## Methods

### Animals, experimental design and sample collection

The study was approved by the Scientific Committee of the Leibniz Institute for Farm Animal Biology (FBN). The experiment was generally licensed and authorized by the ethics committee of the federal state of Mecklenburg-Western Pomerania, Germany (Landesamt für Landwirtschaft, Lebensmittelsicherheit und Fischerei; LALLF M-V/TSD/7221.3–1–053-15).

**Fig. 4:**
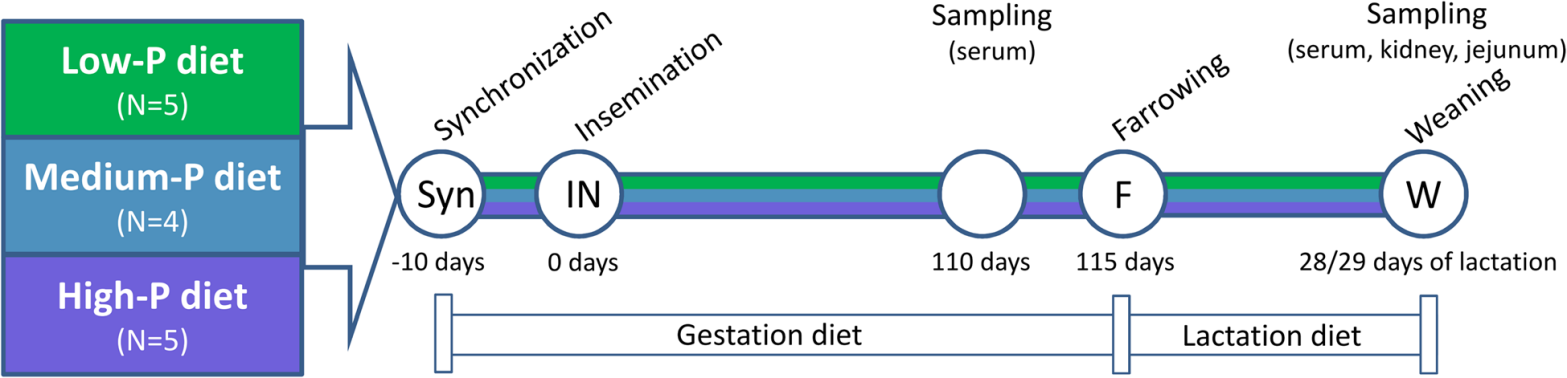
Experimental design of the trial with divergent P supplies throughout gestation and lactation (*n* = 14). Samples were obtained at 110 days of gestation and day 28/29 of lactation

In this study, 14 nulliparous German Landrace sows from the same herd owned by the Leibniz Institute for Farm Animal Biology (FBN) with an age of 11 months were randomly assigned to three dietary groups prior to synchronization (Fig. 4). Sows were fed soy/barley standard diets with medium (M, *n* = 4), reduced (L, *n* = 5) and higher (H, *n* = 5) amounts of dietary P throughout an adaptation period (10 days until insemination), gestation (115 days) and lactation (29 days). Iso-energetic and iso-nitrous dietary formulations for adaptation and gestation were mixed in two batches and contained total dietary P contents of about 0.46% (M), 0.37% (L) and 0.56% (H). Lactating sows received total P contents of 0.61% (M), 0.48% (L) and 0.72% (H). Dietary P contents in diet M corresponded to the current standard feeding recommendations [20]. No phytase was added to the diets. All animals were fed on the same phase feeding regimen (early gestation, late gestation, lactation) and received restricted rations of 2.8 kg up to 6.4 kg to meet the stage-specific requirements. Sows had ad libitum access to water. The sows were kept in two batches, each representing individuals of all three feeding groups. Pregnant sows were kept in group pens supplied with concrete floor. At 110 days of gestation, sows were moved to individual farrowing pens where they remained with their litter throughout the lactation period. No cross-fostering was applied.

Individual body weights were documented prior to synchronization until slaughter (0, 30, 56, 84, 105 days of gestation; day 28 of lactation; carcass weight). Blood samples were taken from *V. jugularis* at 110 days of gestation and at 28 days of lactation after overnight fasting for serum preparation. Veterinary inspection of the carcasses and organs after slaughter confirmed the lack of any impairments, disease symptoms and pathological signs. Sows were anaesthetized by electrical stunning, sacrificed by exsanguination and slaughtered at the Institute’s experimental slaughter facility at day 29 of lactation (corresponds to day 154 on trial) and individual tissue samples were taken. In brief, the left kidney was cut open and cortex was retrieved from the lateral part. Moreover, a section of 10 cm of jejunum was taken at a distance of 200 cm from the stomach. The intestinal tube was opened, the digesta removed and the surface rinsed with ice-cold NaCl (0.9%) to remove residual contaminants. The intestinal epithelium was scraped off with a scalpel. All samples were stored in liquid nitrogen immediately after preparation and stored at − 80 °C until RNA isolation.

### Serum analyses and meat characteristics

Serum minerals including inorganic P (IP), calcium (Ca), magnesium (Mg), and total alkaline phosphatase activity (ALP) were measured using a Fuji DriChem 4000i device (FujiFilm, Minato, Japan). The level of total calcitriol in stored serum samples was measured by an immunoassay using a commercially available kit (AC-62; Immunodiagnostic Systems GmbH, Frankfurt am Main, Germany).

Individual pH of *M. longissimus dorsi* (MLD) and *M. semitendinosus* (MSM) were recorded at 45 min and 24 h post mortem (pH-Star, Matthäus, Pöttmes, Germany). The ash content of MLD samples was determined in triplicate by calcination in a muffle furnace at 600 °C using established protocols [62].

Measurements on zootechnical parameters, serum minerals and post mortem meat characteristics were subjected to a linear model (R language, version 3.6.2, package stats). For the analysis of serum minerals, slaughter batch was included in the model. The post-hoc test of Tukey was used to derive differences between the three experimental groups. The significance level was set at *P* < 0.05.

### RNA library preparation and differential gene expression analysis

Total RNAs were isolated from each of the jejunum and kidney tissue samples of 14 sows fed medium (*n* = 4), lower (*n* = 5) and higher (*n* = 5) level of dietary P by TRI Reagent according to user guides (Sigma-Aldrich, Taufkirchen, Germany), followed by Baseline-ZERO DNase treatment (Biozym, Hessisch Oldendorf, Germany) and purification with the column-based NucleoSpin RNA II-Kit (Macherey–Nagel, Düren, Germany). Concentration of purified RNA samples was measured by Nanodrop 2000 spectrophotometer (Thermo Scientific, Wilmington, USA). The quality of RNA extracts were checked on a Bioanalyzer 2100 (Agilent Technologies, Waldbronn, Germany) yielding RNA integrity numbers (RIN) from 6.3 to 9.1 (average of 8.0). RNA libraries were prepared from the final purified total RNAs according to the TruSeq Stranded mRNA protocol (Illumina, San Diego, CA, United States) and the quality was validated using a Agilent DNA-1000 chip kit (Bioanalyzer 2100). Subsequently, paired-end reads with a length of 2 × 101 bp were generated by RNA sequencing on an Illumina HiSeq2500 instrument. Raw data were quality-checked and pre-processed including the removal of low quality reads (a mean Q-score < 20) and adapters with FastQC v.0.11.7 and Trim Galore v.0.5.0 programs. High quality reads were then mapped to the reference Sscrofa11.1 (Ensembl release 93) and gene features using HISAT2 (2.1.0) [63] and HTSeq 0.8.0 [64] tools. Initial data visualization of gene expression profiles was performed using the mixOmics R package [65]. A sparse Partial Least Squares Discriminant Analysis (sPLS-Da) was used to select the variables (transcripts) with the highest contribution to a component. To account for the experiment including the two tissues and the three experimental groups, in total 4 components were considered in the analysis. Each of these components included 150 variables. Hierarchical clustering of the data was performed using mixOmics with default setting. The differentially expressed gene analysis in the contrast of the three dietary groups (pairwise comparisons) was performed per tissue by the R package DESeq2 v3.4.0 [66]. Very low abundant transcripts having observations in less than four samples were filtered out. The statistical model included information on the mother of the sow to account for the relatedness of pigs. A false discovery rate (FDR) < 0.10 was set as significance threshold to detect differentially expressed genes (DEG) between dietary groups. To assess the differences along the dietary P gradient in the three groups, tissue-specific lists of significant DEGs were combined (132 DEGs for kidney and 79 DEGs for jejunum) and assigned to Kyoto Encyclopedia of Genes and Genomes (KEGG) pathways [Release 93.0, accessed 2/06/2020, reference organism is pig (*Sus scrofa*)] to investigate significant biological alterations using KOBAS 3.0 web server [67]. Pathways were considered significant at FDR (Benjamini and Hochberg)-adjusted P- value ≤0.05. Pathways containing less than 3 input genes and some disease-related pathways that were considered irrelevant were excluded from the KEGG pathway table. The complete results of KEGG pathway enrichment analysis for each tissue are shown in Supplementary Table S2.

## Supplementary information

**Additional file 1: Table S1.** Most differentially expressed transcripts in jejunum between diverging phosphorus diet groups.

**Additional file 2: Table S2.** Results of the KEGG pathway enrichment analysis considering 79 DEGs obtained from analysis of jejunum.

## Data Availability

Raw data are deposited in the ArrayExpress database at The European Bioinformatics Institute (EMBL-EBI, https://www.ebi.ac.uk/arrayexpress/experiments/E-MTAB-9101/,accession number: E-MTAB-9101). RNAseq-reads were mapped to the reference Sscrofa11.1 (Ensembl release 93, https://www.ensembl.org/Sus_scrofa/Info/Index).

## Abbreviations

Calcitriol: 1,25(OH)2VitD3
DEG: Differentially expressed gene
ER: Endoplasmic reticulum
Ca: Calcium
Mg: Magnesium
P: Phosphorus
IP: Inorganic phosphate
ALP: Alkaline phosphatase activity
FDR: False discovery rate
FC: Fold change
H: High P diet group
L: Low P diet group
M: Medium (recommended) P diet group
